# Electrochemical evaluation of the desloratadine at bismuth film electrode in the presence of cationic surfactant: Highly sensitive determination in pharmaceuticals and human urine by Linear sweep-cathodic stripping voltammetry

**DOI:** 10.3906/kim-2101-42

**Published:** 2021-06-30

**Authors:** Yalçın ALTUNKAYNAK, Günay ÖNAL, Abdulkadir LEVENT

**Affiliations:** 1 Department of Chemistry and Chemical Processing Technology, Technical Sciences Vocational School, Batman University, Batman Turkey; 2 Department of Medical Services and Techniques, Health Services Vocational School, Batman University, Batman Turkey; 3 Department of Chemistry, Faculty of Arts and Sciences, Batman University, Batman Turkey

**Keywords:** Desloratadine, voltammetry, bismuth film electrode (BiFE), urine, surfactant

## Abstract

In this study, the electrochemical properties of desloratadine, which is in the second generation antihistamines group, were determined by bismuth film electrode (BiFE) in aqueous and aqueous/surfactant solutions. This compound gave an irreversible and diffusion-controlled reduction peak at about –1.65 V by cyclic voltammetry. It was found that the addition of cationic surfactants (cetyltrimethylammonium bromide (CTAB) increased the reduction current signal of desloratadine, while anionic (sodium dodecylsulfate (SDS) and nonionic (Tween 80) surfactants were found to have an adverse effect. Using linear sweep-cathodic stripping voltammetry, the analytical signal showed a linear correlation with a concentration of 0.1 to 4 µM in 0.04 M Britton–Robinson solution (pH = 8.0) in the presence of 5 mM CTAB, while the detection limit was calculated to be 11.70 nM (3.64 μgL^–1^). This method has been successfully applied for the quantitation of desloratadine in pharmaceutical and urine samples without the need for any separation.

## 1. Introduction

Histamine is a biological amine that is a powerful stimulant of gastric secretion and an effective neurotransmitter. Histamines show their effects by binding to receptors called H1, H2, and H3 in tissues. There are H1 receptors in the ear, nose, and throat area. As the days go by, more drugs have to be used to treat, prevent, and control diseases. Especially, those who want to improve their quality of life are using these drugs. Antihistamines used for ear, nose, and throat diseases are generally divided into two as classical and new generations [1].

Desloratadine (DESL) is a drug derived from loratadine, a second-generation antihistamine commonly used to treat complaints associated with runny nose, sneezing, and allergic rhinitis [2,3]. Besides not affecting the central nervous system, it is not affected by age, sex, and race differences, making DESL more advantageous over other antihistamines [4].

In our literature review, several analytical methods have been reported for the studies on DESL [5–13]. Most of these studies are concerned with the simultaneous determination of Loratadine and DESL, usually using high-performance liquid chromatography (HPLC), electrophoresis, and mass spectroscopy. However, although these techniques show high sensitivity and accuracy in analysis, the disadvantages are as follows: They are expensive devices, the analysis time is long, and the necessity to have expert personnel during the study and for operating the device. Electrochemical methods are more advantageous because of the above stated reasons. In addition, in electrochemical studies, the sample to be analyzed can be analyzed directly without any pretreatment [14–16]. Voltammetric methods can easily meet the needs in this field because they provide safe, accurate, sensitive, and reproducible results in the long term [17–20].

According to our best knowledge, voltammetric studies recommended are summarized in Table 1 for the analysis of DESL in biological fluid and drug. It was stated that DESL gave a reduction peak on the hanging mercury drop electrode (HDME) at approximately –1.48 V in BR buffer (pH = 10.0) [21]. DESL was reduced at –1.55 V in phosphate buffer (pH = 9.0) medium on the glassy carbon electrode (GCE) by cyclic voltammetry (CV) and SWV techniques [22]. In another study, electrochemical properties of DESL was investigated in acetate buffer media with a new sensor designed by adding a mixture of zinc oxide and multiwalled carbon nanotube (ZnO:MWCNT) on the GCE with CV, differential pulse (DP) and SWV techniques [23]. In a similar study, the electrochemical properties of DESL in phosphate buffer (pH = 8.0) media were investigated using LSV technique on modified GCE [24]. Another research group stated in their study that it was oxidized at +1.55 V in phosphate buffer (pH = 4.0) media using the DPV technique on BDD electrode [25].

**Table 1:** Comparison of recommended voltammetric techniques for detection of DESL in the literature.

Electrode	Tech.	Supporting elec.	Linear range (µM)	LOD (nM)	Sample	Ref.
HMDE	SWV	BR(pH 10.0)	1.5-10.0	229	Human Plasma	21
GCE	SWV	PBS (pH 9.0)	25.5–1500	2.75	Pharmaceutical andhuman urine	22
ZnO:MWCNT	SWV	ABS (pH 5.5)	0.02 – 8.0	0.769	Pharmaceutical andhuman urine	23
CMB-MWCNTs/GCE	LSV	PBS (pH 8.0)	1.99–32.9	880	Pharmaceutical andspiked rat serum	24
CP-BDDE	DPV	PBS (pH 4.0)	0.099- 6.3	41	Pharmaceuticals, humanurine and tap water	25
BiFE	LSV	BR (pH 8.0)	0.1–4.0	11.7	Pharmaceutical andhuman urine	This Work

HMDE: Hanging mercury drop electrode. GCE: Glassy carbon electrode. ZnO:MWCNT: zinc oxide and multiwalled carbon nanotube. CMB-MWCNTs/GCE: Glassy carbon electrode modified with carboxymetyl-botryosphaeran and multiwalled carbon nanotubes. CP-BDDE: cathodically pretreated- Boron-doped diamond electrode.

When the relevant studies are examined, the modified and mercury-based electrodes used here are both difficult to prepare and require toxic and expensive reagents/solvents while they are designed. Since mercury toxicity causes environmental pollution and is difficult to transport and disposal, many studies have been conducted on the design for electrode materials alternative to mercury. Among alternative electrodes that do not contain mercury, bismuth exhibits the closest behavior to this electrode [26, 27]. Both in situ and preprepared BiFEs exhibit a very effective stripping behavior in voltammetric studies. This behavior reflects the ability of bismuth to form multicomponent alloys with heavy metals. BiFEs have now proven to be applicable for different target analytes in numerous electroanalytical laboratories [27,28]. The widespread use of BiFE is due to the excellent performance of this electrode for electrochemical properties and analysis of both metals and organic compounds. It is an important advantage that it does not require the removal of dissolved oxygen during stripping analysis and provides the opportunity to work in a wide negative potential range [27–30]. As well as most of the research performed using BiFE has focused on the analysis of metals, there are also organic compound and drug analyzes with recent studies [29–35].

As in other studies, developing a sensitive and selective method in the analytical studies is one of the most important goals. Thus, the modification of electrodes used in electrochemistry is an important parameter to achieve this goal. Therefore, it is extremely important that electrode modification steps are cheap and simple in related studies. The modification process performed with surfactants is quite simple and easy. Also, the benefits of using surfactants in electrode modification stages have been much better understood recently [36].

Surfactants have an enormous impact on today’s chemistry. As a result, their role in electrochemistry has been well documented in recent years [37–39]. Surfactants are substances that tend to deposit at the surface or interface. The precondition for surfactants to be surface active is that these molecules must be adsorbed at the interface between bulk phases such as air/water, oil/water, or electrode/solution. Thus, especially in electrochemical studies, surfactants show the feature of increasing, decreasing or not affecting the electro activity of the target analytic with the phase they form at the electrode/solution interface. As a consequence,, surfactants can change and control the properties of the electrode surfaces. Also, surfactants are known to have the ability to increase or change their reaction velocities [40].

In the literature, it has been found that electrochemical methods are frequently used in the analysis of hydrophilic or hydrophobic drugs, food, biological, herbal, and environmental compounds in solutions containing surfactants [41–46]. Perhaps, considering this information, researchers have examined in detail how the solubility of DESL in aqueous and surfactant media changes depending on pH value [47].

Considering all this information, in this study, the electrochemical properties of DESL were investigated in BR buffer solution containing cetyltrimethylammonium bromide (CTAB) (pH = 8.0) using BiFE. The linear sweep cathodic stripping voltammetry (LS-CSV) method developed for the determination of DESL is simple and fast; it is a very important advantage that BiFE does not contain any toxic chemicals and reagents. The voltammetric method suggested in this study has been successfully applied to drug and urine samples.

## 2. Experimental

### 2.1. Chemicals

DESL (D1069) standard substance was obtained from Sigma-Aldrich (Sigma-Aldrich Corp., St. Louis, MO, USA) and syrup formulation Deloday Sanovel (containing 0.5 mg/mL DESL) was obtained from the pharmacy. DESL (1 mM) was prepared in methanol. Britton-Robinson (BR; pH = 2.0–10.0), phosphate buffer (PBS; pH = 2.0, 3.0, 7.4, 9.0) and acetate buffer (ABS; pH = 4.8) solutions were used as supporting electrolytes. Solutions of stock surfactants (0.1 M) were prepared in anionic (sodium dodecylsulfate, SDS), nonionic (polyethylene glycol octyl-phenyl ether, Triton X-100/TRT) and cationic (Cetyl trimethyl ammonium bromide, CTAB) water, respectively. Bismuth standard (76668) was obtained from Sigma-Aldrich (St. Louis, MO, USA). The desired pH values were adjusted with 5 M NaOH and 5 M HCl solutions. Deionized water from the Milli-Q system was used in the preparation of the solutions. All solutions were stored at +4 ^°^C and experiments were carried out at room temperature.

### 2.2. Materials

The electroanalytical studies were performed using the Autolab PGSTAT 128N (EcoChemie company) and a Bioanalytical System Inc. (BAS), electrochemical workstations containing a three-electrode cell unit. Electrochemical analysis was controlled with Nova 1.11 software. The cell stand has three electrode systems: GCE as working electrode (BAS MF 2012), platinum wire (BAS MF 1032) as counter electrode; Ag/AgCl saturated KCl (BAS MF 2052) as reference electrode was used. pH measurements were made using WTW Inolab pH 720.

### 2.3. Preparation of BiFE

Every day; before starting the experiments, the surface of the GC electrode was mechanically cleaned to obtain reproducible and sensitive results. For this purpose, Al_2_O_3_ (alumina, Ø: 0.01 μm, diameter), which was made into a slurry with water, was poured on the polishing kit (BAS MF 1040) and the electrode surface was polished by circular movements on this slurry. The electrode surface was then gently cleaned with distilled water and then cleaned in an ultrasonic bath for 5 min.

BiFE has been prepared according to the references [26–28]. While the ABS (pH = 4.8) solution containing Bi (III) was mixed at a constant speed (600 rpm), a constant potential of –0.9 V was applied to the GCE for 120 s. Then, the electrode was transferred to another cell and voltammetric measurements were recorded in the supporting electrolyte solution. During the study, electrochemical cleaning technique was applied between each measurement to clean the GCE surface from Bi. For this process; a clean surface is obtained by applying a constant potential of + 0.8 V for 30 s to the electrode in ABS (pH = 4.8). Stripping conditions are as follos: deposition potential = –0.8 V, deposition time = 120 s. Linear sweep voltammetry (LSV) parameters: step potential = 0.00244 V, scan rate = 0.1 V/s.

### 2.4. Voltammetric method

CV and LS-CSV measurements were recorded in different supporting electrolyte mediums containing surfactant in the range of 0 to –2.2 V after the preparation of the BIFE described in section 2.3.

### 2.5. Sample preparation

Deloday syrup (0.5 mg/mL DESL) obtained from pharmacies was used in the application of the proposed method. After the syrup was thoroughly mixed, the voltammetric cell containing the supporting electrolyte selected for optimum conditions was added at the level of µL of this syrup, and so voltammetric measurements were carried out as described in sections 2.3 and 2.4. The sample solution was analyzed directly, and quantifications were carried out by means of the calibration curve method from the calibration equations.

The urine sample was obtained from a healthy volunteer who did not smoke or use drugs. The samples (10 mL urine sample spiked with working solution of 20 µM DESL) were centrifuged at 5000 rpm for 10 min in order to remove unexpected endogenous chemicals. 300 µL of the urine sample from the upper clear part was diluted to 10 mL with the selected supporting electrolyte [BR (pH = 8.0, containing 5 mM CTAB)]. Same procedure process was applied to nonspiked samples to obtain blanks. The determination of DESL in spiked urine samples was carried out after its standard additions of 0.2, 0.3, 0.4, 0.5, and 0.6 µM. Electrochemical measurements were performed as described in section 2.3 and 2.4. Recovery studies were carried out using the multiple standard addition method. Measurements were repeated three times.

## 3. Results and discussion

### 3.1. Cyclic voltammetry

**Figure 1:**
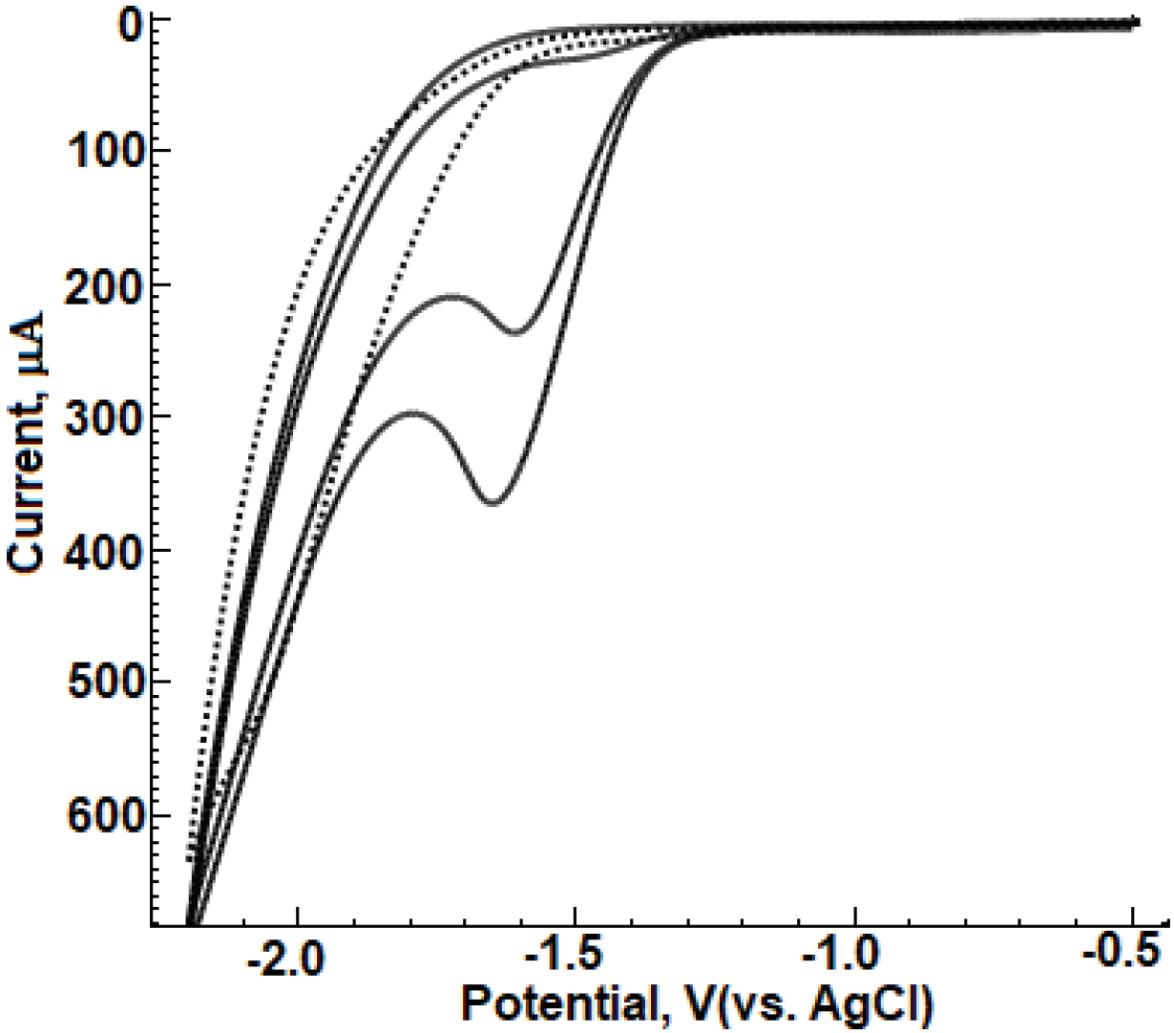
Cyclic voltammograms of 0.1 mM DESL in 0.04 M BR (pH = 8.0, containing 5 mM CTAB) medium, with BiFE. Scan rate:0.1 mV/s. Dashed line, supporting electrolyte.

CVs of 0.1 mM DESL were recorded in BR buffer (pH = 8.0 containing 5mM CTAB) using BiFE electrode in the range of 0 to –2.2 V. Two-cycle CVs of DESL are presented in Figure 1. In the 1st and 2nd cycles, the reduction peak potentials occurring at about -1.63 V and -1.59 V, respectively, and their current signals were also observed at 166 µA and 100.7µA. In the literature, when the reduction peak potential of DESL was pH> 9, it was recorded as -1.48 V with CV technique on HDME in BR medium [21]. In another study, when GCE was used, an irreversible reduction peak current of DESL was obtained at about –1.55 V in PBS (pH = 9.0) medium [22]. In our study, when the reduction peak potential obtained in the medium of BR (pH = 8.0, containing 5mM CTAB) on the BiFE surface is compared with the results in the related studies, it is seen that DESL is reduced at more negative potentials. As seen in Figure 1, as the number of cycles increases on the BiFE surface, the decrease in the reduction peak signal intensity indicates the possibility of adsorption of the reduction products on the electrode surface.

When the data obtained from the relationship between the potential scan rate in the range of 12.5–700 mVs^–1^ and the peak current are evaluated; Linearity in the **Ip/** ν relationship [ **Ip** (µA) = 0.3114 *ν* (mV/s) + 54.174, r = 0.9956] and the slope obtained from the **LogIp** / **Log** ν relationship [ **LogIp** = 0.4416 **Log** ν + 1.1217, r = 0.9921] is close to 0.5. It shows that the electrochemical process taking place on the BiFE surface in BR (pH = 8.0, 5 mM containing CTAB) medium can be diffusion-controlled adsorption.

**Figure 2:**
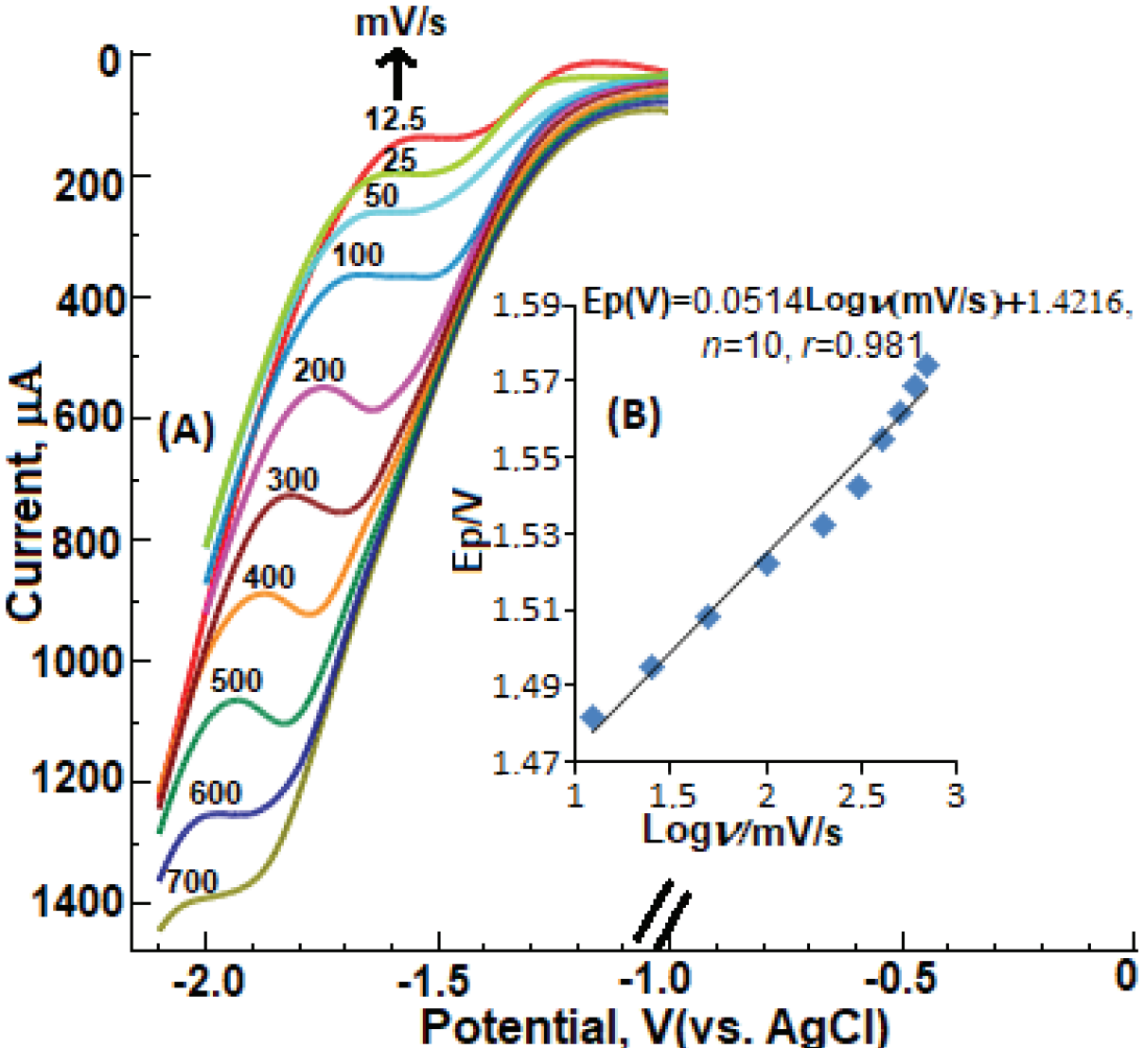
LSV curves for 0.1 mM DESL on the BiFE electrode at different scan rates in 0.04 M BR (pH = 8.0, containing 5 mM CTAB).

On the other hand, as illustrated in Figure 2; as the scan rate increased in the 12.5-700 mVs^-1^ range, the reduction peak potential shifted to a more negative region. This phenomenon is characteristic for an irreversible or semireversible electrochemical reaction [48]. **Ep** /Log **Log** ν relationship illustrated in Figure 2 can be expressed as follows in the potential scan rate range studied; E **_p_** (V) = 0.0514 **Log** ν (mV/s) + 1.4216.

For an irreversible electrode process, the relationship between **Ep** /Log **Log** ν is defined as the following [ *E*_p_= *E* ^0^+(2.303RT/α *n* F)log(RT *k* ^0^/α *n* F) +(2.303RT/α *n* F)log *ν* ] [49]. In the equation, α is the charge transfer coefficient and n is the number of electrons transferred in the redox reaction. R (8.314 J K^–1 ^mol^–1^), T (298 K) and F (96480 C mol^–1^) are known constants. The slope value in the **Ep** /Log **Log** ν relationship is 0.0514. Using the equation above, the value of αn is calculated as 1.147. In the case of the fully-irreversible electrode, it can be accepted as α = 0.5 for most systems. Thus, a value of *n* =2.29 (≈ 2) is obtained. The number of 2 electrons obtained on the BiFE electrode was found to be compatible with those in the literature [21, 22].

### 3.2. Optimization of the BiFE

**Figure 3:**
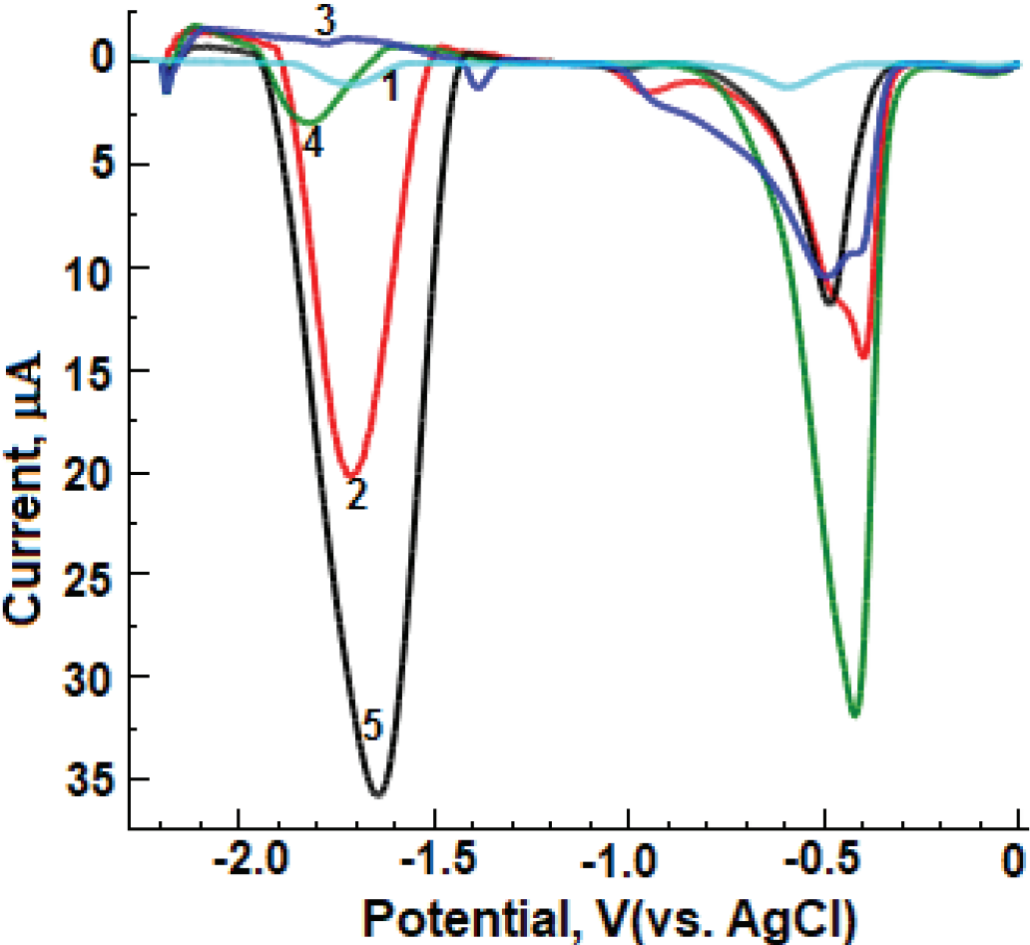
LS-CSV vurves in 0.04 M BR (pH = 8.0) medium for 60 μM DESL; (1) GCE, (2) BiFE, (3) BiFE+5 mM SDS, (4) BiFE+5 mM TRT and (5) BiFE+5mM CTAB. BiFE conditions are as follows. Deposition potential: –0.9 V, deposition time: 120, stripping conditions: deposition potential: –0.8 V, deposition time: 120 s. LSV parameters are as follows. Step potential: 0.00244 V, scan rate: 0.1 V/s.

As can be seen clearly in Figure 3, it has been observed that the analytical signal is approximately ten times more sensitive with the formation of a bismuth film on the GC electrode surface. Thus, the optimization study of BiFE was realized in order to obtain the most sensitive analytical signals on the electrode. First, as explained in section 2.3, Bi^3+^ concentration was carried out for the film electrode formed on the GC electrode surface using the ex-situ technique. Voltammograms were recorded in the range of 1–10 ppm at –0.9 V/120 s in ABS (pH = 4.8) medium, in BR (pH = 8.0) buffer medium at a constant DESL concentration of 60 µM. The most suitable and sensitive analytical signals were obtained at 6 ppm. Deposition potential was practiced in the range of 0 to –1.4 V under conditions where the Bi^3+ ^concentration was kept constant at 60 ppm and the deposition time for 60 s. Optimal results were obtained at –0.9 V deposition potential. The deposition time (0–210 s) was performed by keeping the Bi^3+^ concentration (60 ppm) and the deposition potential (–0.9 V) constant. The significant and sensitive reduction peaks were obtained at 120 s. Optimum conditions for BiFE design in the next stages of studies was selected as concentration (6 ppm), deposition potential (–0.9 V) and deposition time (120 s). 

### 3.3. Effect of surfactant

After the electrochemical reduction study of DESL on BiFE in aqueous solutions, the effect of CTAB, sodium dodecylsulfate (SDS) and polyethylene glycol tert-octylphenyl ether (TRT) surfactants on DESL’s electrochemical behavior were investigated under optimum conditions. For this purpose, as given in Figure 3, the voltammograms of DESL (60 µM) were recorded on the BiFE using the LS-CSV technique in a BR buffer (pH = 8.0) solution containing surfactant (5 mM). As stated in Section 3.2, it was observed that the bismuth film formed on the GCE surface had a very catalytic effect in the direction of the reduction of DESL. By continuing to work in the light of this information, as can be observed from Figure 3, in the case of aqueous solutions, reduction behavior of the DESL at the BiFE (at –1.72 V) is advantageous over that observed at the bare GCE (at –1.74 V), as the reduction peak potential is close to each other and the peak current is about ten times higher. Especially, in the case of CTAB, reduction behavior of the DESL at the BiFE/CTAB (at –1.65 V) is advantageous over that observed at the BiFE/SDS (at –1.78 V)/BiFE/TRT (at –1.83 V), as the peak potentials are about 0.13 V/0.18 V positive, and the peak currents are about eighteen/six times higher, respectively. Addition of CTAB resulted in a marked increase of DESL peak intensity on BiFE, suggesting that surfactant media alters the over potential of the electrode reaction and increases the electron transfer rate between DESL and electrode surface, as explained above. It is well known that surfactants are adsorbed at the electrode-solution interface and consequently act as modifying agents. At the same time, this structure takes place on the electrode surface where it greatly influences the electrochemical pathway of the analytic. Probably, there was no positive electrostatic attraction between DESL and related surfactants on the BiFE surface in the presence of SDS and TRT surfactants in the BR(pH = 8.0) medium. Furthermore, DESL is protonated when pH < 8, taking into consideration that its pKa = 8.65 [50] and pKa_1 _= 4.41/pKa_2 _= 9.97 [47] values in aqueous medium. As a result of this, the fact that the lowest analytical signal was obtained on the BiFE surface in anionic surfactant (SDS) medium indicates that there is no electrostatic attraction on the electrode surface between protonated DESL and SDS. On the other hand, as DESL approaches its neutral character towards the pH value, the maximum analytical signal intensity on the BiFE can be explained by a different mechanism called “coadsorption” [51,52]. This mechanism can generally occur in the presence of cationic surfactant (CTAB) and it has been understood to be compatible with the literature [33].

In the next stages of the study, optimum CTAB concentration (in the range of 0.5–10 mM) was performed. It was observed that the reduction peak currents of DESL increased on the BiFE surface up to a concentration of 5 mM. It was observed that the peak currents remained constant as the concentration increased and then the peak current intensity decreased.

### 3.4. Effect of pH value

**Figure 4:**
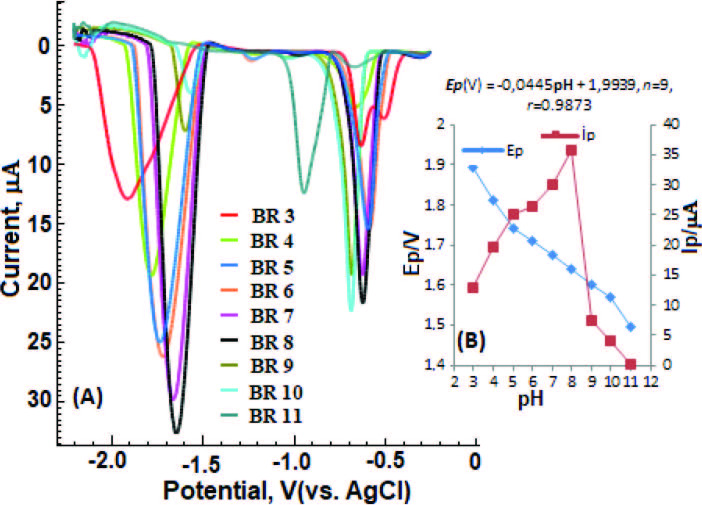
LS-CSV curves obtained by BiFE of 60 μM DESL in 0.04 M BR (pH = 3.0–11.0) buffer solutions: 5 mM CTAB, BiFE, stripping and LSV parameters are given in Figure 3.

**Figure 5:**
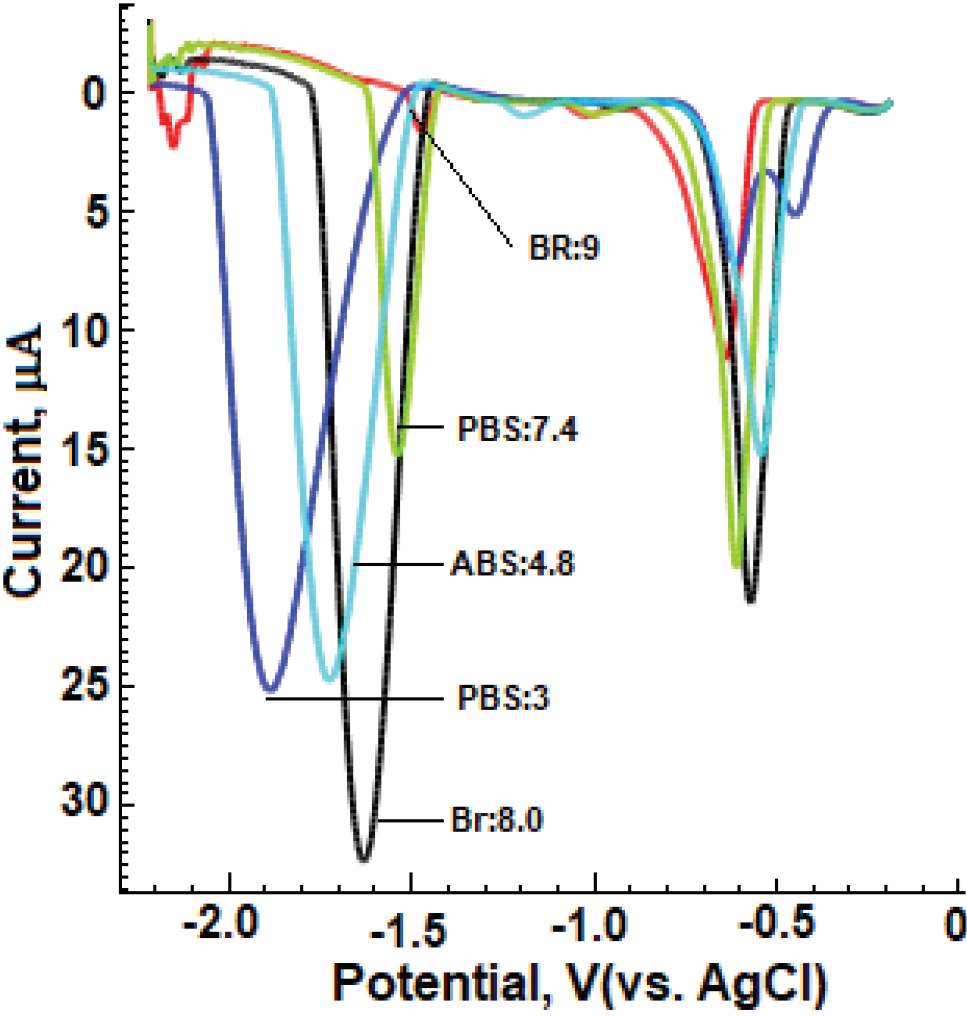
LS-CSV curves of 60 μM DESL obtained by using BiFE at different supporting electrolytes and p values. Surfactant: 5 mM CTAB, BiFE, stripping and LSV parameters are given in Figure 3.

The effect of different supporting electrolyte and pH value on the electrochemical behavior of DESL (60 µM) was recorded LS-CS voltammograms in the range of +0.0 V to –2.2 V (Figure 4). For this purpose, 0.1 M ABS (pH 4.8), 0.1 M PBS (pH 3.0, 7.4 and 9.0) and 0.04 M BR buffer (pH 3–12) solutions including 5 mM CTAB were used as supporting electrolytes. As can be clearly seen in Figure 4, the highest analytical signal and well-defined peaks were obtained in BR (pH = 8.0). In addition, when Figure 5 is examined in detail, it is seen that even if different supporting electrolyte and pH environments are used, the BR (pH = 8.0) medium is proven to be the most suitable medium.

It was observed that as the pH value increased, the cathodic peak potential shifted to more positive values. When the relation of DESL Ep [Ep (mV) = –44.5pH + 1993.9 r = 0.9873)] realized on the electrode surface in the electrochemical process is examined; The slope value (–44.5 mV / pH) in the studied pH range was obtained. 

The evolution of Ep with pH shows a linear [ **Ep** (mV)=-44.5 **pH** +1993.9]. The slope was observed from pH = 3.0–11.0 with a negative slope of 44.5 mV per pH unit (r = 0.9873), which is close to the theoretical value of 59 mV/pH. According to these results, it is seen that the proton contribution in the reduction process is equal to the number of electrons. This result was found to be consistent with other studies in the literature [21–25]. Also, the evolution of Ep with pH shows two almost linear segments. The intersection point of the curves (∼pH 4.0–5.0 and pH 8.0–9.0) are close to the pKa_1 _= 4.41[47] and pKa_2 _= 8.65[50], respectively. 

### 3.5. Optimization of deposition parameters

As stated in Section 3.1, the accumulation potential and accumulation time parameters of the proposed method were carried out since the electrochemical process taking place on the BiFE surface has diffusion-controlled adsorption effect. With this in mind, the most sensitive and symmetrical analytical signals of 10 µM DESL solution were studied in BR (pH 8.0 /5 mM CTAB containing) medium. First, the accumulation potential was studied in the range of 0 to –1.4 V under conditions where the deposition time was kept constant for 60 s. The most sensitive and symmetrical analytical signals were obtained under conditions where the deposition potential was –0.9 V. Furthermore, the most sensitive and smooth peaks were obtained at 120 s in the study of the effect of the deposition time on the reduction peak in the range between 0 and 210 s under conditions where the deposition potential was kept constant at –0.9 V.

### 3.6. Optimization of linear sweep voltammetry (LSV) parameters

Since sensitivity is an important parameter in analytical method development studies, the step potential (1 mV/10 mV) and scan rate (12.5/700 mV / s) parameters, which increase the sensitivity of the voltammetric method developed in this study, were studied to improve the intensity and shape of the analytical signal.

The most sensitive and significant analytical signals on the BiFE of 10 µM DESL were reached at 5 mV step potential under optimum operating conditions where the scan rate was kept constant at 10 mV/s. Under conditions where the step potential was kept constant at 5 mV, the most sensitive and symmetrical reduction peaks were obtained at a scan rate of 200 mVs^–1^. In all subsequent stages of the studies, it was decided to continue with the optimum conditions for LS-CSV as step potential 5 mV and scan rate 200 mVs^–1^.

### 3.7. Analytical application of voltammetric method

**Figure 6:**
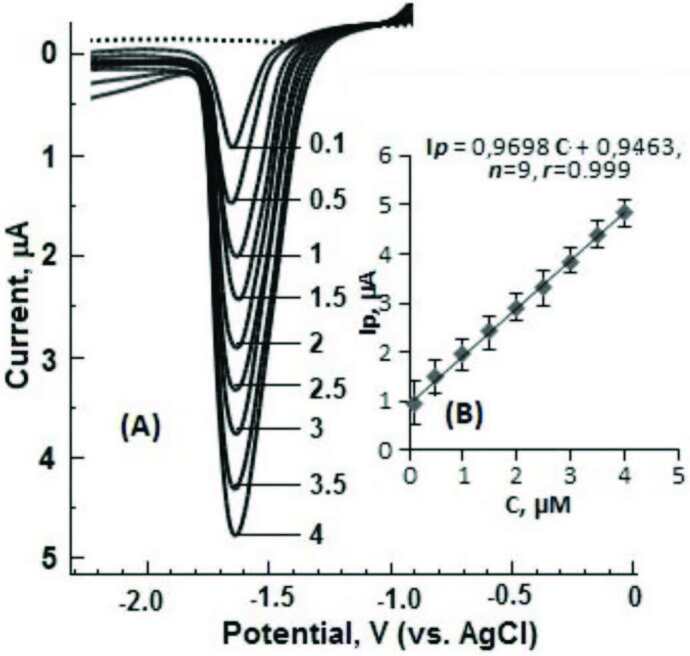
LS-CSV response of BİFE for different DESL concentrations in 0.04 M BR (pH = 8.0, containing 5 mM CTAB). Dashed line, supporting electrolyte. BiFE, stripping, and LSV parameters are given in Figure 3.

In order to determine the analytical performance properties of the voltammetric method developed on the BiFE, the analytical working range, sensitivity, repeatability, and selectivity conditions of the method were studied under optimum conditions. The LS-CSV curves of DESL in different concentrations were recorded in optimum experimental conditions. The well-visible reduction peak voltammograms and corresponding calibration curves at –1.65 V potential using BiFE in BR buffer (pH 8.0, containing 5mM CTAB) of standard DESL solutions in the concentration range of 0.1–4.0 µM are shown in Figure 6.

As can be seen from Figure 6, it was observed that the analytical signal increased regularly as the DESL concentration increased successfully in the range of 0.1–4.0 µM. The equation obtained by plotting the concentration versus current values [ **Ip** (µA) = 0.9698 **C** (µM) + 0.9463, ( *n* = 9)] appears to have a good linearity with a correlation coefficient of 0.999.

The analytical sensitivity of the voltammetric method developed on the BiFE was calculated according to the limit of detection (LOD = 3s/m) and the limit of quantification (LOQ = 10s/m). In the equation, (s) is the standard deviation of the nine smallest signal responses that can be read on the line of the supporting electrolyte solution, and (m) is the slope on the calibration curve. As a result of the calculations, LOD and LOQ values were found as 11.70 nM (3.64 µgL^–1^) and 39 nM (12.12 µgL^–1^), respectively. The studies in Table 1, which summarizes the voltammetric methods recommended for the analysis of DESL in different mediums, and the results obtained in this study were compared in terms of LOD values. Although HMDE’s [21] LOD value is low, it is also not recommended to be used in studies due to the toxicity of the mercury electrode. Furthermore, cleaning the mercury before each analysis and the harm of the waste mercury to the environment can be counted among the disadvantages of this study. Although the results obtained with ZnO: MWCNT [22] and CMB-MWCNTs/GCE [24] are similar, the preparation process and cost of modified electrodes are important for scientific studies. Using bare GCE [22] a low LOD value was obtained, but this value found is far from the analytical working range. Although the LOD value obtained with CP-BDDE [25] is close to the result obtained in our study, the cost of the method increases due to the electrode used. In addition to features such as environmentally friendly, cheap, easy to prepare and good analytical performance of the BiFE used as electrode in this study, the low LOD value obtained are important advantages.

The precision of the developed voltammetric method was assessed by the intra-day and inter-day repeatability of the cathodic peak current values. It was repeated seven times for LS-CSV technique on the same day and on different days in five different solutions under optimum conditions. Relative standard deviations (RSD%) of 2 µM DESL in BR buffer (pH = 8.0/5 mM CTAB containing) medium in terms of intra-day cathodic peak current values were found to be 1.25% and 1.78%, respectively. Repeatability results inter-days under the same conditions were found as RSD 1.45% and 1.94% in terms of cathodic peak potential and peak current values, respectively. According to the results, it can be said that the repeatability of the cathodic peak potential and peak current values obtained at the BiFE containing CTAB with the LS-CSV technique is very good.

**Figure 7:**
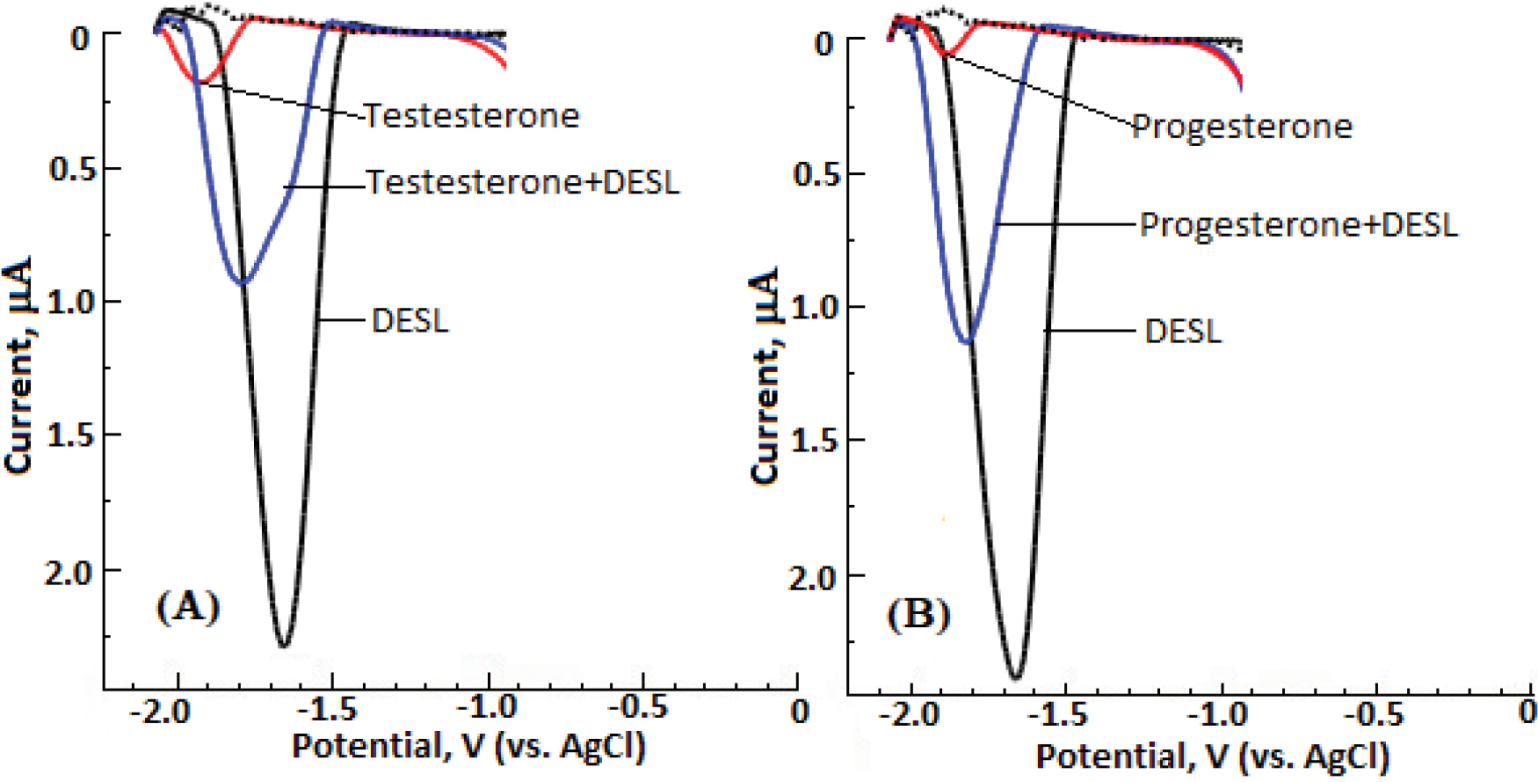
LS-CSV curves of DESL(2.0 μM) in the presence of (A) testesterone and (B) Progesterone at their 100-fold excess. Dashed line, supporting electrolyte. BiFE, stripping, and LSV parameters are given in Figure 3.

In this study, because the sensitivity and precision of the proposed method are very good, the selectivity study of the method has been tested on substances likely to be found in biological and pharmaceutical samples. By keeping the DESL concentration constant at 2.0 µM, the interfering species (Na^+^, Mg^2+^, Ca^2+^, P^3+^, Fe^3+^, Co^2+^, Cu^2+^, Ni^2+^, uric acid, α-tocopherol, folic acid, ascorbic acid, dopamine, epinephrine, norepinephrine, testosterone, estradiol, and progesterone) concentration was kept at a rate of 100 times and the study was carried out. The tolerance limit was accepted as ±5%. It was observed that among these substances, especially testosterone (Figure 7A) and progesterone (Figure 7B), interfered with these working conditions and other substances did not. There are already studies of testosterone [33] and progesterone [53, 54] at BiFE. It can be said that the method developed in this study is very selective for the DESL determination of BiFE under optimum working conditions.

As stated above, since the sensitivity and selectivity of the proposed method are very good, the accuracy of the related method was tested in pharmaceutical and urine samples by performing recovery experiments. Voltammetric studies of syrup samples prepared by the way described in Section 2.5 were carried out under optimum conditions of the proposed method. When voltammograms belonging to syrup samples and standard DESL were checked against, it was observed that the curves were compatible. After each successful syrup sample addition, the recorded analytical signal was placed in the relevant calibration curve and the results found were calculated as recovery. When the results given in Table 2 are examined, it can be said that the precision and accuracy of BiFE containing CTAB very good for detection of the DESL in the syrup samples with a valid recovery between 94.75% and 106.60%.

**Table 2:** Voltammetric method analysis findings in syrup containing DESL.

Sample	Found (µM)a,b	Recoveryb % + RSD%
1	0.477	95.50 + 3.28
2	0.484	96.87 +3.14
3	0.473	94.75 + 3.24
4	0.533	106.60 + 2.64
5	0.517	103.40 + 2.21

a Deloday syrup (contains 0.5 mg / mL DESL).b Results are the average of three analyzes.

**Figure 8:**
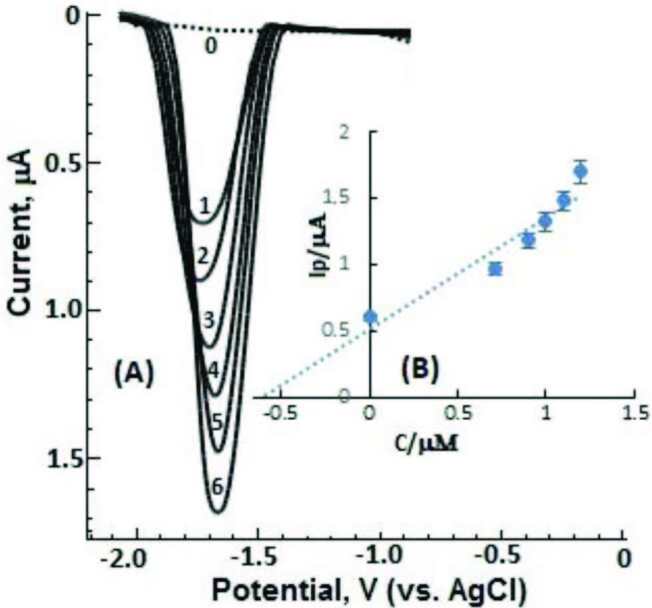
LS-CSV curves of urine sample; (0): Blank urine, (1): spiked with DESL to reach the final concentrations of 0.6 μM, after standard additions of (2):0.2, (3): 0.3, (4): 0.4, (5): 0.5 and (6): 0.6. BiFE, stripping and LSV parameters are given in Figure 3.

Due to the high sensitivity and reproducibility of the electroanalytical method developed at the BiFE containing CTAB for DESL determination, selectivity conditions were investigated in complex matrix media (containing water, salt, and biomolecules) such as urine sample by using the multiple standard addition method. As explained in Section 2.5, after voltammetric analysis of pure urine sample was performed with BiFE under optimum conditions, standard DESL additions were successfully made on the spiked urine sample, and voltammograms were recorded. The LS-CS voltammograms obtained are shown in Figure 8. The reduction peak observed at about –1.65 V increased consistently after each successful standard DESL addition. After plotting the analytical signal values for each added DESL concentration, a linear correlation was obtained [ **Ip** (µA) = 0.8404 **C** (µM) +0.508, n = 6, *r =* 0.991]. By using the multiple standard addition method, recovery % and RSD % values were calculated. According to the results represented in Table 3, it is seen that the electroanalytical method developed at the CTAB-BiFE can be successfully applied to urine samples.

**Table 3:** Analysis of DESL in spiked urine samples by LS-CSV using GCE.

Added (µM)	Found (µM)a	Recovery (%) ± RSD (%)
0	0.60	± 2.86
0.2	0.81	106.5 ± 2.97
0.3	0.92	107.2 ± 3.06
0.4	0.97	93.6 ± 2.71
0.5	1.08	95.8 ± 2.95
0.6	1.25	108.1 ± 3.24

aValues reported are the average of three independent analyses of each spiked sample.

## 4. Conclusion

In this study, an accurate, sensitive, selective, fast, economical and simple voltammetric method was developed for the determination of DESL in the pharmaceutical and urine samples with BiFE in BR buffer (pH = 8.0, containing 5 mM CTAB) medium. When the LOD and linear working range results obtained for the analysis of DESL at the BiFE in the real samples with the LS-CSV method in the cationic surfactant medium are compared with the results of the studies summarized in Table 1. It has been observed that the developed voltammetric method has more sensitive and advantageous results. As can be seen in the studies presented in Table 1, it has been determined that mercury and modified electrodes were used in the proposed methods for the electrochemical properties and analysis of DESL. The toxicity and disadvantages of mercury electrodes in electrochemical studies are also known. In addition, taking into consideration that the process of modifying an electrode, it is also a well-known fact that it has a process that requires more time and effort along with higher cost than the use of a bare electrode. In the light of this information, it can be said that this electroanalytical study performed using BiFE containing CTAB in the LS-CSV technique is more advantageous for the determination of DESL in pharmaceutical drugs and urine.
